# From labels to influencers: evaluating the alignment of nitrate and beetroot juice claims on sports supplement marketing with scientific consensus

**DOI:** 10.3389/fnut.2026.1886906

**Published:** 2026-07-15

**Authors:** Pedro Estevan Navarro, Cristina González-Díaz, Alejandro Perales, David Romero-García, Antonio Molina-López, Ángel Gil-Izquierdo, Isabel Sospedra, José Miguel Martínez-Sanz

**Affiliations:** 1 Faculty of Health Sciences, University of Alicante, Alicante, Spain; 2 CTS-595 Research Group, Department of Computer Science, Pablo de Olavide University, Seville, Spain; 3 Communication and Social Psychology Department, Faculty of Economics and Business, University of Alicante, Alicante, Spain; 4 Communication, Food and Consumption (FOODCO) Research Group of the University of Alicante, Alicante, Spain; 5 Communication Sciences and Sociology, Faculty of Communication Sciences, Rey Juan Carlos University, Madrid, Spain; 6 Optics, Pharmacology and Anatomy Department, University of Alicante, Alicante, Spain; 7 Real Madrid Graduate School, Faculty of Sport Sciences, Universidad Europea de Madrid, Madrid, Spain; 8 Quality, Safety, and Bioactivity of Plant Foods Group, Department of Food Science and Technology, CEBAS-CSIC, University of Murcia, Murcia, Spain; 9 Nursing Department, Faculty of Health Sciences, University of Alicante, Alicante, Spain

**Keywords:** advertising claims, beetroot, labeling, nutrition, sports supplement

## Abstract

**Background:**

Dietary nitrate supplementation, commonly delivered through beetroot-based products, is widely used in sports contexts due to its proposed ergogenic effects. However, within the European Union, no authorized health claims exist for nitrate supplementation, raising concerns about the accuracy of marketing messages. In the current digital landscape, commercial communication strategies increasingly incorporate advertising techniques such as influencer endorsement, which may further shape consumer perceptions and behaviors.

**Methods:**

An observational cross-sectional study was conducted to evaluate nitrate- and beetroot-based dietary supplements marketed in Europe. Products were identified through online platforms, and data regarding labeling, recommended dosage, claimed effects, and advertising strategies—including the use of influencers and endorsements—were systematically collected. Claims were assessed for their compliance with current scientific evidence and international consensus recommendations.

**Results:**

A total of 18 products were included. Most supplements presented performance- and health-related claims despite the absence of authorized claims in the European Union. While 61.1% of products reported nitrate dosages within recommended ranges, overall compliance with evidence-based protocols was limited. Only a minority of claims aligned fully with scientific consensus. Notably, 55.6% of products incorporated celebrities or influencers as part of their marketing strategy, whereas none presented external scientific endorsement. References to supporting scientific evidence were scarce and often incomplete.

**Conclusion:**

Nitrate- and beetroot-based supplements marketed in Europe frequently display inconsistencies between scientific evidence and commercial claims. The widespread use of influencer-based marketing, combined with the absence of external endorsement, may contribute to the dissemination of potentially misleading information. These findings highlight the need for stricter alignment between scientific evidence and digital marketing practices to support informed consumer decision-making and reduce potential health misinformation in the context of sports nutrition.

## 1 Introduction

The International Olympic Committee (IOC) defines sports food supplements (SFS) as “a food, food constituent, nutrient or non-food component that is purposefully ingested as part of the normal diet for the purpose of eliciting a specific health or performance effect.” (1). SFS are widely consumed to complement nutritional intake, and products marketed as sports food supplements represent a growing segment of this market, particularly among physically active individuals and athletes (2–5).

Currently there is a high intake of SFS by physically active individuals and athletes (6, 7), 40%−100% of athletes of any level (amateur, professional, and elite) consume them (4) and for the industry this constitutes its main commercial target (3). Consequently, several thousand sport supplements products are currently available on the market (4, 5).

Nitrates are normally found in vegetables, and these are the best option for human consumption such as spinach, beets, and rocket (8). In recent years, interest has increased in the possible effects of improving performance and post-exercise recovery after the consumption of nitrates, since one of its main mechanisms of action is that after taking it, the nitric oxide generated can improve blood flow during physical exertion, reduce oxygen demand and improve mitochondrial function, among other physiological impacts (9).

Nitrates are a very promising supplement but are underused in most sports and individual cases. Furthermore, they have been less studied, compared to other supplements both in the general population and in elite athletes, such as creatine or caffeine, either due to lack of knowledge or due to the protocol for their use (2, 3, 10). This variability in effective dosages and supplementation protocols emphasizes the importance of clear, evidence-based nutrition-related information. However, as highlighted in recent analyses on other widely consumed ergogenic aids such as caffeine and creatine, it is also essential to examine how these supplements are presented to consumers through advertising messages and product labeling, since health and performance claims often do not fully align with the scientific consensus or established protocols (11, 12). This justifies the need for research that systematically evaluates the adequacy of the claims made for nitrate-based supplements (NBS) in relation to the available scientific evidence. Specific laws and regulations in the European Union (EU) regulate aspects such as labeling, advertising and the truthfulness of the information provided to consumers (13). Specifically focused on supplements for sports people and athletes, researches, and institutions such as the Australian Institute of Sport (AIS) provide advice, recommendations on how to use and consume, as well as dosage, protocols, safety, precautions and warnings for these (2).

One of the key elements in the advertising communication of food supplements (SFS) is the use of Health Claims, defined in Regulation (EC) 1924/2006 as “any statement on labels, advertising or other marketing products claiming that the consumption of a particular food may benefit health.” (14). The European Food Safety Authority (EFSA) is the body responsible for the scientific assessment of applications for claims, issuing opinions that allow the European Commission to decide on their approval. The authorized claims, together with their conditions of use, are listed in a Community register of Health Claims (14). In the specific field of sport, EFSA also analyses applications for physical performance claims, rejecting those without conclusive scientific evidence (14, 15). Nevertheless, it should be noted that only a limited number of claims have been approved, and that some supplements with strong scientific support, such as nitrates, have neither been evaluated by EFSA nor have any health claims approved by the EU.

For the purposes of this study, we distinguish between four categories of statements found in product labeling and digital communication: (i) authorized health claims—statements formally approved by EFSA under Regulation (EC) 1924/2006 and listed in the EU Register; (ii) marketing claims—promotional statements made by the manufacturer that do not correspond to any EFSA-authorized formulation; (iii) physiological or performance-related statements—descriptions of mechanisms of action or ergogenic effects supported by scientific consensus documents (e.g., IOC, AIS, and ACSM) but not formally authorized by EFSA; and (iv) unsupported statements—claims backed by neither regulatory authorization nor scientific consensus. Throughout the manuscript we use the generic term “claims” as shorthand for all four categories, and specify the subtype where relevant.

In parallel with the growth of the sports supplement market, the digitalization of food environments has transformed how nutrition-related information is communicated to consumers. Online platforms, including brand websites and social media, have become primary channels for disseminating product-related claims, often combining scientific language with persuasive marketing strategies (16, 17).

In this context, the use of influencers and athletes as promotional agents has become increasingly common in sports nutrition marketing. These figures can shape consumer perceptions by increasing the credibility and attractiveness of products, even when the underlying scientific evidence is limited or not clearly communicated (17, 18). This is particularly relevant in the case of ergogenic aids such as NBS, where efficacy depends on specific protocols, dosages, and contextual factors that are not always adequately reflected in commercial communication.

Therefore, beyond the evaluation of labeling compliance, it is necessary to examine how advertising strategies—especially those involving influencers and digital marketing—may contribute to the misalignment between scientific evidence and the information presented to consumers.

Although current European legislation requires that marketing communication on dietary and sports supplements be clear, truthful, and not misleading (13), compliance in digital environments remains uneven, as product information is often intertwined with persuasive marketing strategies that do not always reflect current scientific evidence (16, 17). Irregularities have been documented in the advertising and labeling of widely consumed sports supplements such as protein, creatine, or fat burners (18), and although fraud in NBS is comparatively less frequent, discrepancies between declared and actual nitrate content have also been reported (19). Despite the growing relevance of influencer-based promotion in the sports nutrition sector, limited research has systematically examined how these digital marketing practices coexist with the scientific validity of nutrition-related claims displayed on supplement labels and official websites.

These concerns are not exclusive to the European context. Previous studies, conducted mainly in the United States, the United Kingdom, and Canada, have documented serious problems in the marketing of dietary supplements, including misleading advice provided in health food shops, exaggerated or inaccurate claims in mass-media advertising, and the use of multi-level marketing structures that combine personal testimony with commercial incentives (20, 21). Comparing these findings with the European context—where the regulatory framework is, in principle, more restrictive, but enforcement of digital communication remains heterogeneous—is therefore relevant to identify both shared patterns and region-specific challenges in sports supplement marketing.

The aim of this study was to assess the compliance of nutrition-related claims and recommended dosages of nitrate- and beetroot-based dietary supplements marketed in Europe with current scientific evidence and international consensus recommendations. Additionally, the study aimed to describe the advertising strategies used in the promotion of these products, including the presence of influencers, celebrities, and other digital marketing elements, and to explore their coexistence with potentially non-compliant or unsupported claims.

We hypothesized that nitrate- and beetroot-based supplements marketed in Europe would frequently present claims and recommended protocols not fully aligned with current scientific evidence, and that influencer-based promotion would coexist with claims lacking adequate scientific support.

## 2 Methods

### 2.1 Study design

An observational and cross-sectional study was carried out based on the content analysis of the different claims included in the commercial communications of a sample of nitrate and beetroot juice supplements, analyzing these claims in the light of the main international consensus and scientific evidence in this area, following the methodology of previous research carried out by the same research team (11, 12, 22). For terminological consistency, throughout the manuscript we use the term “nitrate-based supplements” (NBS) as the umbrella category, encompassing both products formulated as concentrated nitrate sources (capsules, powders, and gels) and beetroot juice-based products (shots, liquids) marketed as ergogenic aids for sports performance. The term “sports food supplements” (SFS) is reserved for the broader market category defined by the IOC consensus statement (1), and “ergogenic aid” is used only for general conceptual references. In addition, the study design, as well as the development of the manuscript, followed the STROBE statement (23).

### 2.2 Product search strategy

The search for the sample products was conducted in August 2024 through Amazon and Google Shopping because they are the main online shopping websites. Moreover, we added the sports supplements shown on the Informed Sport platform which is considered one of the most important platforms for certificated brand supplements with an anti-doping seal (24). To carry out the search process, the keywords used were “Nitrates” or “beetroot juice”, which filtered by European region.

In all the platforms, the initial search was carried out on the first 10 pages after applying the inclusion criteria, if there were not 10 pages of results, the search was carried out on all the results obtained. Subsequently, we redirected to each of the websites of the selected supplement brands to obtain the claims for each of them. The process of obtaining each component of the sample was different depending on the portal visited.

The first 10 pages of results were screened on Amazon and Google Shopping using the default platform ranking (“relevance”), with the search terms “nitrates” or “beetroot juice” and the regional filter set to Europe. To minimize algorithmic personalization based on prior searches or purchase history, all searches were performed in incognito/private-browsing mode without any prior account history. We acknowledge, however, that platform ranking algorithms are proprietary and not fully transparent, and that exact reproduction of the ranking cannot be guaranteed across users, geographical locations or time points.

These platforms were selected not only because of their commercial relevance, but because they represent the digital environments in which consumers—particularly young and physically active populations—are most frequently exposed to integrated marketing messages combining product information, influencer endorsements, and persuasive branding strategies.

### 2.3 Inclusion criteria

Supplements in “capsule”, “pill”, “powder”, “liquid”, “shot”, or “gel” form, with nitrates or beetroot juice as the sole active components and they mentioned claims of the website and labeling information, and whose product information was available in English, Spanish, French, Italian, German, or Portuguese (the languages mastered by the research team).

### 2.4 Exclusion criteria

Supplements containing nitrates or beetroot juice, but combined with other active components such as arginine, caffeine, citrulline malate, or tart cherry, products whose information was available only in a language not mastered by the research team; and products whose official website or technical sheet was not accessible at the time of the search.

### 2.5 Data extraction and coding

After the search process, a content analysis of the selected products was conducted. We carried out a systematization by applying standardized parameters to the selection of Saldaña's contents (25). An *ad hoc* checklist (see Table 1) was designed and approved by the research team taking into account the nature of the object of study. The checklist was developed based on the previous experience of the authors in sports supplement labeling research and on their published work analyzing health and performance claims in similar contexts. An expert consensus process was conducted within the research team to ensure content relevance and completeness.

**Table 1:** Definitions of study variables.

No.	Variable	Definition
1	Name of the supplement	Word designating or identifying it as a trade name
2	Corporate brand	Name of the company manufacturing the supplements
3	Health Claims (EFSA) or Claims	If the supplement has a health claim in the labeling approved by the European Commission according to the opinion report of the European Food Safety Authority (EFSA). Authorized and non-authorized health claims can be found at Register of Health Claims https://ec.europa.eu/food/food-feed-portal/screen/health-claims/eu-register
4	Ergogenic and physiological effects (51)	Whether the effects stated in the labeling are in line with the actual and indicated effects through consensus documents of scientific reference institutions in sports nutrition and/or systematic reviews with or without recent meta-analyses.
5	Ergogenic and/or physiological effect or claim as described by the brand/company and number of claims	Ergogenic and/or physiological effects described by the company in the labeling of the product and which do not fit in the provided 3 and 4.
		Number of declarations
6	Dosage indicated by EFSA, reference institutions or company	EFSA stated dose
		Dose indicated by reference institutions and/or systematic reviews with or without meta-analysis
		Recommended dosage as stated by the company on the labeling
7	Protocol indicated by EFSA, reference institutions or company	Protocol indicated by EFSA
		Protocol indicated by reference institutions and/or systematic reviews with or without meta-analyses
		Protocol indicating labeling as recommended
8	Format presented by the product	Format in which the product is presented
9	Sports involved (3)	Describes the sports in which their use is recommended.
10	Bibliography	Describes relevant scientific literature such as consensus articles, systematic reviews, meta-analyses etc
		Indicate the type of article for each of the indicated ones
		Year of publication of each of the items indicated
		Indicate the reference or the link to access the article
11	Adverse effects	Describes possible adverse effects according to the manufacturer's indications (protocol and dosage)
		Which ones?
12	Food safety	Indications of restrictions or side effects on its consumption for certain population groups
		Which ones?
13	Allergens (52)	Indications the presence/traceability of allergens
	Indicate the list of allergens
14	Advertising claim	They use a form of celebrity or influencer on the website or product labeling. Note: We understand a famous person as someone who has gained public recognition due to their professional talent in various fields such as film, music, sports, etc. In literature, they are also referred to as “celebrities”
		Does the influencer have training and/or experience with the content of the supplement? Note: As for an influencer, although there is no universally agreed-upon definition, we refer to individuals who have established their recognition and notoriety through their publications on digital platforms. In this way, influencers accumulate followers by sharing their personal and everyday lives, which serve as the basis for their recommendations and advertising of products, services, and lifestyles (53)
15	External endorsement	Presents as endorsement a scientific institution or association or others
		Which ones?
16	Antidoping	Any type of anti-doping seal that guarantees that the product or batch is free of doping substances is described or indicated
		Which ones?
17	Patented ingredients	Any patents used in the composition of the product are described or indicated.
		Which ones?
18	Scientific advice	Indication of the person or persons who carry out the scientific advice at a nutritional level, indicated on the website as staff, collaborators or other sections

The variables were operationalized to allow dichotomous observation (presence/absence) on the product website and labeling, minimizing subjective interpretation during coding phase (26). Prior to the analysis, a pilot test was conducted following Krippendorff's recommendations (27). This step enabled: (1) identifying items requiring refinement in the checklist; and (2) confirming the clarity and comprehensibility of the recording instructions (28). The checklist was not formally validated due to the exploratory and descriptive nature of the study; however, its content validity was strengthened through expert consensus and pilot testing. The checklist included variables related to dosage, health and performance claims, scientific references, warnings, allergen labeling, and marketing strategies (Table 1).

Additionally, specific variables related to advertising and marketing strategies were included, such as the presence of influencers, athletes or celebrities in product promotion, the use of persuasive communication elements, and references to external endorsements or certifications. These variables were incorporated to better understand how digital marketing practices may influence the presentation and interpretation of supplement-related claims.

Data extraction followed a consensus-coding design. Two researchers (P.E.N. and J.M.M.-S.) independently coded every product against the closed set of dichotomous (presence/absence) variables defined in the checklist. Every item on which the two coders differed was subsequently reviewed jointly with the wider research team, discussed against the operational definitions, and resolved until full (100%) agreement was reached for all products and all variables. Because coding disagreements were resolved through deliberation rather than retained as independent parallel codings, conventional inter-coder reliability coefficients (Cohen's kappa, Krippendorff's alpha) were not computed in this first application of the instrument; the implications of this choice are addressed in the Limitations section. The complete operational version of the checklist is provided as Supplementary material (Supplementary Tables S1, S2) to allow independent assessment and replication of the instrument.

## 3 Results

### 3.1 Product identification

**Figure 1:**
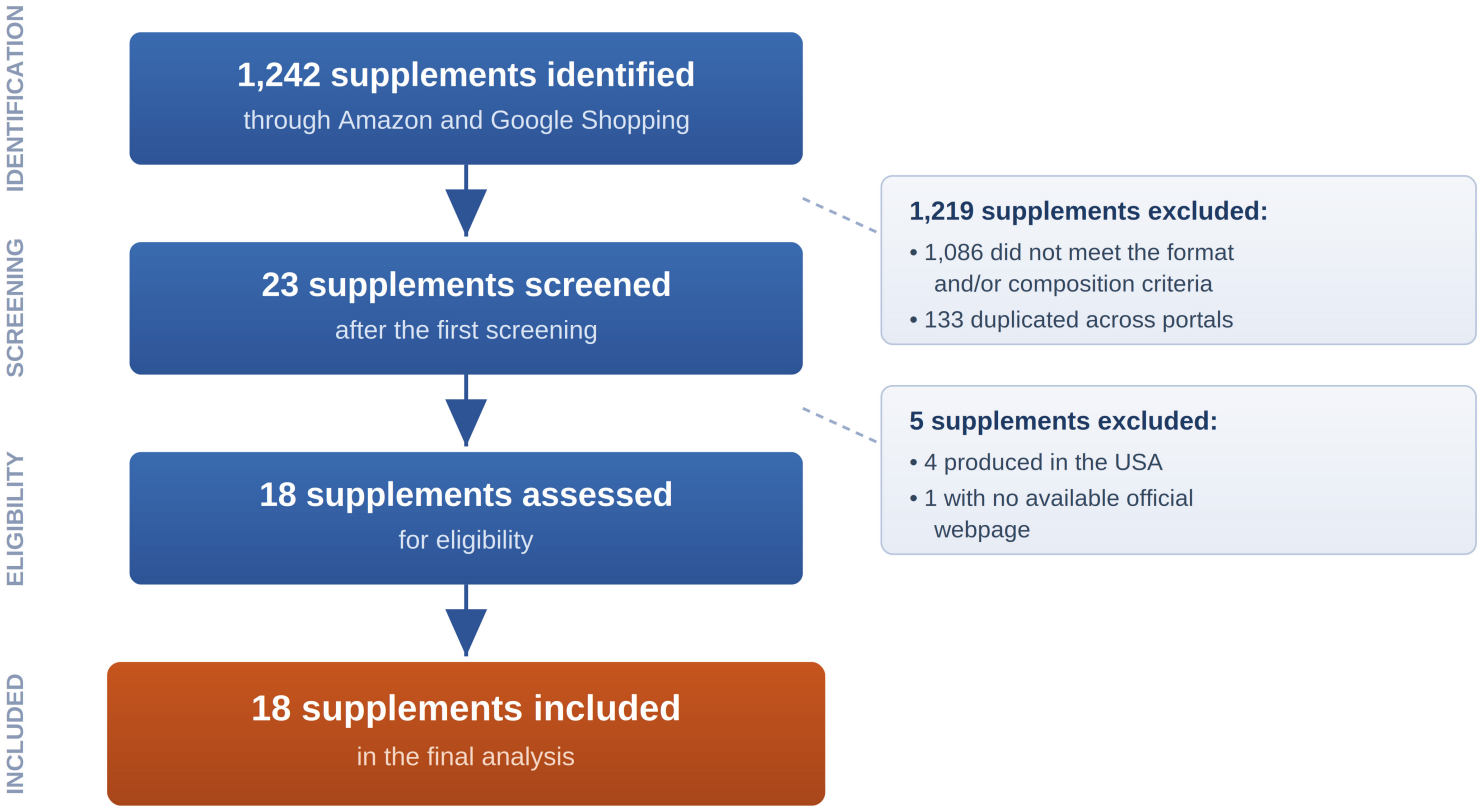
Flow diagram of the product search and selection process. From an initial 1,242 results, 1,086 were excluded for not meeting the format and/or composition criteria and 133 for duplication across one or both commercial portals, leaving 23 records after the first screening. A further 5 products were excluded (1 produced in the USA and 4 without an available official webpage), resulting in 18 supplements included in the final analysis.

In the search, 1,242 results were obtained, of which 18 nitrates or beetroot juice supplements belonging to different trademarks met the inclusion criteria established in the methodology. Hence, 1,219 results were rejected: 1,086 for not meeting the format and/or composition criteria and 133 for appearing repeatedly on one or both of the commercial portals (Figure 1). After the first screening 23 results were obtained, but 5 products were rejected: one because it comes from the USA and four because the official web page was not available. For the final 18 selected supplements, the dose, use, protocol and other relevant information were specified in the technical sheet or official web page, and these products were subsequently analyzed using the predefined checklist.

### 3.2 Claims, product dosage and compliance with current scientific evidence

As can be seen in Tables 2a, 2b. regarding the Nitrates dose of the products, 5.6% (*n* = 1) of them contained < 300 mg per dose, 61.1% (*n* = 11) contained 300–500 mg per dose, 5.6% (*n* = 1) contained >500 mg per dose, and 27.8% (*n* = 5) did not specify the dose per serving. In addition, Table 3 establishes a percentage distribution of each healthy property declaration found in the NBS sample and further compares the proposed dosages and the type of healthy property declaration indicated by the manufacturer in each nitrates sports supplement. The health declaration most frequently described for the nitrates NBS was “increase the blood flow” in 44.4% of the sample.

**Table 2a:** Claims aligned with scientific consensus documents.

Claims by scientific literature/ institutions	Number and percentage of total supplements for which this claim is made	In line with scientific opinion and institutions. Yes or PARTIALLY^*^
Improving sporting performance	5 (27.8%)	YES
Improves exercise tolerance and efficiency	1 (5.6%)	YES
Contributes to increased athletic performance	2 (11.1%)^*^	PARTIALLY^*^
Helps to improve oxygen consumption efficiency and endurance	4(22.2%)^*^	PARTIALLY^*^
Contributes to improving performance more efficiently	1 (5.6%)^*^	PARTIALLY^*^
Optimizes sports performance	1 (5.6%)^*^	PARTIALLY^*^

Own elaboration based on the search data. The “In line with scientific opinion” column refers exclusively to alignment with the formulations used in international scientific consensus documents on NBS (1, 2, 29–31) and is independent of EFSA regulatory authorization; at present, no health claim for nitrate or beetroot juice has been authorized by EFSA. Operational criteria: YES—the claim formulation, terminology and conditions of use (dose 300–500 mg; timing ~2–3 h pre-exercise) match those described in at least one consensus document. PARTIALLY—the claim describes a consensus-supported effect but uses different wording or spelling, or does not specify the conditions of use.

**Table 2b:** Claims made by brand or company not aligned with scientific consensus.

Claims by brand or company	Number and percentage of total supplements for which this claim is made
Promotes muscle contraction and accelerates recovery	3 (16.7%)
Improves blood flow	8 (44.4%)
Contributes to improved aerobic performance	2 (11.1%)
Contributes to the elevation of blood flow, increasing the supply of oxygen and nutrients to cells	3 (16.7%)
They will contribute to an increased supply of oxygen and nutrients to the cells.	3 (16.7%)
Promotes muscle recovery	1 (5.6%)
Regulates the immune system, brain, arteries, liver, pancreas, uterus and lungs.	2 (11.1%)
Increases exercise endurance	1 (5.6%)
Improving cardiovascular health	1 (5.6%)
Improving cognitive function in young adults	1 (5.6%)
Improves endurance performance	2 (11.1%)
Increases oxygen efficiency	2 (11.1%)
Reduces muscle fatigue	2 (11.1%)
Optimal performance for longer	2 (11.1%)
Improves sprint performance	2 (11.1%)
Improve Vo2max	1 (5.6%)
Improves muscle efficiency	1 (5.6%)
Improves the ability to perform repeated sprints	1 (5.6%)
Supplements without health claims on the label or official websites	5 (27.8%)

Own elaboration based on the search data. None of the claims listed here correspond to formulations authorized by EFSA under Regulation (EC) 1924/2006 or recognized in international consensus documents NBS (1, 2, 29–31).

**Table 3:** Distribution of nitrates supplement characteristics about publicity, antidoping test, patent, and research staff.

Characteristic	% of supplements that present	% of supplements that do NOT show
They use a form of celebrity or influencer on the website or product labeling.	55.6% (*n* = 10)	44.4% (*n* = 8)
Do it present an external endorsement?	0% (*n* = 0)	100% (*n* = 18)
It has a doping stamp	50% (*n* = 9)	50% (*n* = 9)
Does it have a patent among its ingredients?	5.6% (*n* = 1)	94.4% (*n* = 17)
Does the company indicate which experts advise them?	22.2% (*n* = 4)	77.8% (*n* = 14)

Only 38.9% of the supplements did comply with the protocols established by the literature, among those who did not comply with the protocols we found that the most common reason was that they made a mistake in the time to take the pre-exercise supplement, stating that they had to take it 1 h before exercise instead of 2 h. The most common format was “shot” with 38.9% (*n* = 7), followed by “powder” with 27.8% (*n* = 5), “capsule” with 16.7% (*n* = 3), “liquid” with 11.1% (*n* = 2) and finally “tablet” with 5.6% (*n* = 1).

Only 22.2% (*n* = 4) of the supplements studied mentioned the sports in which effects have been observed and there is scientific consensus supporting their use in them, of which one or two sports out of all accepted sports were mentioned. Only one supplement brand mentioned more than 50% of the sports for which consensus and research bodies claim that the use of nitrates would produce positive performance effects.

Regarding marketing strategies, more than half of the analyzed supplements (55.6%) incorporated the use of celebrities or influencers as part of their promotional communication. In contrast, none of the products presented external institutional endorsement supporting their claims. Additionally, 50% of the supplements highlighted anti-doping certification, primarily through recognized seals such as Informed Sport.

Of the supplements analyzed, only 11 (61.1%) presented the traceability list or the list of allergens visibly. Of those that did present it, it was found that the most common allergens were: crustaceans, soy, and milk, being present in four products (36.4%); followed by eggs, fish, and mollusks present in three products (27.3%) and finally sulfur, present in two products (18.2%). Several products claimed not to have any type of allergen.

In terms of bibliographic references, only 16.7% (*n* = 3) mentioned scientific studies, of which none were systematic reviews with or without meta-analyses or consensus articles. The year of publication of the studies was >2005.

Only 16.7% (*n* = 3) state adverse effects/warnings for special populations, being in all cases “do not use in pregnant women”, although without really arguing the reason, nor evidence to support this statement.

Notably, the presence of influencer-based marketing strategies frequently coexisted with claims that were not fully aligned with current scientific evidence, suggesting a potential imbalance between promotional appeal and evidence-based communication.

### 3.3 Influencer-based advertising strategies and scientific support

Of the 18 products analyzed, 10 (55.6%) used celebrities or influencers in their commercial communication. Among these, the most frequent figures were professional athletes and sports clubs sponsored by the brand. In contrast, none of the products presented external scientific or institutional endorsement supporting their claims. Anti-doping certifications were displayed by nine products (50.0%), most commonly the Informed Sport seal (*n* = 7; 77.8% of those with certification). Only four products (22.2%) disclosed the scientific staff advising the company, who were predominantly dietitians-nutritionists and/or sport science professionals.

When the sample was examined according to the presence of influencer-based marketing, products using influencers (*n* = 10) and those without (*n* = 8) displayed broadly similar patterns in terms of dose specification and anti-doping certification, and the use of claims not aligned with scientific consensus was observed across both groups. Given the descriptive design and the small subgroup sizes, these patterns are reported qualitatively and were not subjected to inferential statistical testing.

## 4 Discussion

### 4.1 Product selection

In this study, we analyzed the claims, dosage, protocols and advertising characteristics of a sample of NBS. We found that the most commonly used dosage is between 300 and 500 mg, which follows the recommendations of the main scientific bodies and consensus documents (1, 2, 29–31).

The most frequently presented claim in the sample of NBS was “improves blood flow”, which was present in 44.4% of the products analyzed. Among the claims recognized by the main scientific bodies and consensus documents (1, 2, 29–31), the most widely used in our sample was “improves performance” (27.8%). Several supplements presented claims that were only partially aligned with these recognized statements, with variations in wording or spelling. It would be advisable for these brands or products to use the correct terminology.

The use of NBS in different sports has been shown to improve performance (1, 2, 29–31). There are several brands that use these supplements for the effects they produce, although we have observed that they rarely specify the sport for which these supplements are beneficial (only 22.2% of the sample studied mentioned any of the sports correctly). Brands usually recommend a dose that is in line with the evidence in most cases (61.1%), in products intended to reach this dose in a single intake (shots, tablets, etc.). In other cases, the nitrate content is either not clearly stated, or is outside the recommended range (1, 2, 29–31).

In addition, in some sports such as football, these supplements are widely used although in a very irregular way in terms of protocol and control of variables of interest such as the nitrate content of the food, it is still necessary to work on education so that the quantities, protocols, etc. are respected in the teams to guarantee their effect as well as guaranteeing the reference bodies such as the AIS (2, 32).

### 4.2 Adverse effects of nitrates consumption and lack adequate scientific support

In our sample, 16.7% of the supplements analyzed stated some kind of warning/adverse effect regarding the intake of nitrates, always being “do not use or take in pregnant women” Side effects of nitrate intake are uncommon. Some studies have reported pink urine color in up to 72% of participants (33), an unpleasant taste in 56% (8) and gastrointestinal discomfort in 32% (34), although these effects depend on individual perception by the athlete (32). However, no study in our review identified specific warnings regarding pregnant women or problems related to pregnancy.

Across the 18 products analyzed, we identified 25 different performance- and health-related statements about the effects of NBS. Only two of these were aligned with the formulations used by the main scientific bodies and consensus documents (1, 13, 29–31). Unlike caffeine or creatine—for which EFSA has issued scientific opinions and the European Commission has reached decisions on specific claims—no EFSA scientific opinion, whether favorable or unfavorable, has ever been issued on nitrate or beetroot juice supplementation. As a result, no claim for these substances has ever reached the approval stage at the European Commission. It is also noteworthy that, for nearly a decade, no substantial regulatory developments or newly authorized health claims have been introduced in the specific field of sports food supplements (13, 14, 35). This creates a paradoxical situation in which sufficient scientific evidence supports the ergogenic use of nitrate, yet the absence of any regulatory framework for its claims means that the way these products may legally be advertised must be assessed against general advertising legislation rather than against substance-specific health-claim regulation. Only the statements “Improves sports performance” and “improves exercise tolerance and efficiency” were considered to meet the criteria of the institutions and bodies (1, 2, 29–31). As long as the dosage was adequate, between 300 and 500 mg.

It is important to underline that scientific support and regulatory authorization operate as two distinct dimensions in our analysis. Several physiological and ergogenic effects of NBS are supported by consensus documents and meta-analyses (1, 2, 10, 29–31), yet no health claim related to nitrate has been formally authorized by EFSA. Conversely, EFSA authorization of a claim implies a specific regulatory procedure, but its absence does not in itself indicate a lack of scientific support. When we describe a claim as being “in line with scientific opinion”, we refer exclusively to alignment with scientific consensus formulations; we do not imply regulatory authorization. This situation places manufacturers of NBS in a regulatory gray zone: the products have recognized ergogenic effects in the scientific literature, but there is no authorized wording they can legally use to communicate these effects, which may partly explain the heterogeneous and sometimes overstated claims observed in our sample.

None of the supplements are supported by external endorsement, for example by the Spanish College of Cardiologists, which would lend weight to the claim of “improving cardiovascular health” if it were in line with the existing evidence. It seems clear that there is a clear dose-response relationship between the amount of nitrate consumed and the concentration of nitrates and nitrites in the blood (10, 36); but the quantities and timings described in the literature and in this study respect the pharmacokinetics to maximize the effect at the indicated time (10).

### 4.3 Use of influencers and implications

The widespread use of influencers and elite athletes in the promotion of NBS raises additional concerns regarding the quality and reliability of the information provided to consumers. A particularly relevant aspect of our findings is that, although 55.6% of the products analyzed incorporated celebrities, athletes or sports clubs in their digital marketing, none of these figures were identified as registered dietitians-nutritionists, sport scientists, or professionals with verifiable training in nutrition or health sciences.

Given the strong persuasive power of these social media strategies, particularly among younger and physically active populations, this type of communication has the potential to influence purchasing decisions and consumption behaviors (17, 18); however, direct causal evidence is beyond the scope of an observational study such as ours, and consumer-level research is required to establish the magnitude and direction of this effect. This situation highlights the need for stricter regulation not only of product claims but also of the actors involved in their promotion, ensuring that nutrition-related information is delivered by qualified professionals and aligned with current scientific consensus.

This promotional approach often coexists with claims that are not fully supported by scientific evidence, raising concerns about the potential for misleading interpretations among consumers. Currently, the volume of advertisements and commercial communications attributing performance enhancements to certain products, including SFS to which athletes are exposed, is of a large dimension. Authorities and consumer associations should require that these claims and/or advertisements relating to nutrition and health as well as sports performance effects are supported by scientific evidence, expert or consensus bodies to avoid possible confusion or misleading consumers, exaggerating the performance enhancing capacity or effect of a certain product or simply claiming effects that are not in line with current evidence (3, 37, 38). Some authors have stated that athletes often do not have all the necessary information and/or the information they have is wrong, about the uses, effects, etc. of sports supplements, so that its intake should always be supervised by a professional. In addition, it has also been observed that there is a great deficiency in basic and general nutritional knowledge about nutrition and sports practice, both for NBS and other supplements in both amateur and elite sportsmen and women (20, 32). This lack of knowledge could be due to or even aggravated by the knowledge or beliefs that are transmitted by figures close to the athlete such as influencers, friends, family, coaches, and training partners. Figures who do not necessarily have knowledge of the area, but are given great credibility and trust by the athlete (3, 39).

At the same time, no product presented external scientific endorsement, revealing a marked imbalance between persuasive authority and formal qualification. This pattern is consistent with previous research showing that influencer-based promotion of dietary supplements on Instagram tends to omit critical information on dosage, adverse effects and contraindications, while amplifying suggestive product names and unsupported promises of efficacy (40), and that nutrition messages delivered by athletes and content creators on digital platforms frequently rely on personal beliefs or commercial interests rather than on scientific evidence (41). In our sample, claims such as “improves blood flow”, “regulates the immune system, brain, arteries, liver, pancreas, uterus, and lungs” or “improves cognitive function in young adults” coexisted with this type of endorsement, despite not corresponding to any claim authorized by EFSA or supported by consensus documents on NBS (1, 2, 29–31). These observations underline the importance of ensuring that nutrition-related communication on sports supplements is delivered, or at least supervised, by qualified professionals, given the strong modeling effect that athletes and influencers exert on physically active populations.

### 4.4 Poor or incomplete information in advertising and potential consumer confusion

Advertising for foods and supplements often fails to provide sufficient additional information for safe and effective use, such as effects, dosages, protocols, contraindications, and target populations. Critically, many corporate websites, technical data sheets, labels, or other commercial communication do not include accessible bibliographic references that substantiate the claims made, thereby reducing the transparency and scientific grounding of the information presented. When such references are absent or not readily available, consumers may struggle to evaluate the credibility of the claims and may be exposed to confusing or misleading messages.

Beyond informational gaps, the presence of banned or undeclared substances has also been reported an issue of vital importance under the World Anti-Doping Agency (WADA) framework, since it can jeopardize athletes' health and careers (17). To mitigate this risk, the display of third-party anti-doping or doping-free seals is relevant. In our sample, several supplements carried such seals, with Informed Sport being the most recognized and respected at the European level (13).

In our sample, only 16.7% of the supplements studied showed correctly cited bibliographic references, although in no case were they international reference and/or consensus articles, and in all cases, they were not linked, simply cited at some point on the web page in bibliographic reference format, which would make them difficult to use for the average non-academic consumer. This is in line with other studies which have found that in many cases the information is misleading or confusing and can be counterproductive (20). Other authors have noted that the bibliographic citations did not correspond to the statements attributed to him in at least 50% of the cases studied (42, 43). These types of bad practices are also observed in other types of dietary supplements and foods (44). We have observed in our sample a low degree of adequacy in terms of statements, protocols, sports in which the effect is had… indicated by the manufacturer, the statements were partially correct or inadequate or did not correspond to the type of supplements studied, which could cause errors or confusion among consumers.

Our findings extend previous evidence from the United States, the United Kingdom, and Canada (20, 21) to the European context, showing that the structural problems of supplement marketing—exaggerated claims, absence of evidence references, and amplification through trusted figures—are present even within a more restrictive regulatory framework, with the added specificity that digital communication and influencer-based promotion now play a prominent role.

### 4.5 Action and proposals to deal with incomplete, inaccurate, or confusing claims

In the context of digital nutrition information, the combination of incomplete scientific evidence and persuasive marketing strategies may contribute to the dissemination of potentially misleading messages, particularly among non-expert consumers. This is especially relevant given the increasing influence of social media on dietary behaviors and supplement use. All health legislation must ensure that communications are truthful and do not mislead users, are subject to the latest scientific evidence and are always authorized by health authorities. With food and SFS, the criteria established by WADA must be respected (17).

In this regard, recent regulatory activity in Spain illustrates this gap. In June 2026, the Spanish Council of Ministers approved the draft of a new Anti-Doping Act, which incorporates a specific section dedicated to doping prevention, including education and awareness measures for athletes, families and sports organizations (45). However, this regulatory effort addresses doping prevention—a context in which sports supplements are relevant as a potential source of inadvertent contamination—but does not regulate the advertising or health-related claims of these products, which remain governed only by general advertising legislation.

Some authors have pointed out the regulatory difficulty of these products, which is nonetheless necessary because several studies have found fraudulent labeling, especially with protein, creatine, or weight loss supplements, which may be unintentional or voluntary adulteration or some kind of cross-contamination (21, 46, 47). While it is true that NBS fraud is not as common, there are some discrepancies between nitrate content and labeling (18, 19), are products within the same niche market of Sports Supplements, so their regulation should not be neglected, and further studies and analyses should be carried out to check their suitability.

On this context, in Spain, the health and consumer authorities are responsible for regulating food advertising. In addition, the legislation is tough in its fight against misleading or deceptive advertising (48). The courts, the advertising sector and the media have an out-of-court resolution body, the Association for the Self-Regulation of Commercial Communication (Autocontrol), the Association of Communication Users (AUC) defends the rights of citizens against the media, giving them the possibility to report any advertising content that is considered illegal. This imbalance may have implications beyond individual consumer protection. Populations with limited access to qualified nutrition professionals, lower digital literacy, or stronger reliance on social media as a primary source of dietary information could be disproportionately affected by misleading or unsupported supplement claims. In this sense, the asymmetry between scientific evidence and digital marketing of sports supplements may not be a neutral phenomenon and could potentially contribute to existing health inequities in the digital nutrition environment, although this hypothesis would require direct empirical testing in consumer-level studies before firm conclusions can be drawn. Therefore, it is of utmost importance that quality and truthful advertising is required, safe for the consumer and adapted to current evidence, thus guaranteeing quality and fair advertising of the products (49).

### 4.6 Study limitations

One of the limitations of the study is that only supplements that had been as an active ingredient were studied, leaving out of the analysis some interesting NBS that contained other agents such as citrulline or arginine. Another limitation is that there were supplements that did not offer the information required for the study. The products that were based on beet juice that were sold as juice, but not as a supplement, it was expected that the labeling would not provide the information necessary for the study. Even so, this study focuses on the European context and the most recent or most important evidence, thus paving the way for future research in this field, studying, for example, supplements with combinations of ingredients and/or outside the European context. The authors have used the term “claims” but it should be clarified that it is a term of European legislation indicated by EFSA (14, 15). These claims are based on consensus studies, systematic reviews with meta-analyses and most current evidence, but NBS do not have any claims approved by EFSA at present (14, 35, 50). It should also be noted that the checklist used in this study was developed *ad hoc* and has not been formally validated. Although its content validity was strengthened through expert consensus and pilot testing, its generalization to other supplement categories or regulatory contexts should be interpreted with caution. Future studies should consider the formal validation of this checklist and its application to other categories of sports supplements and wider geographical contexts.

In addition, although Amazon, Google Shopping, and Informed Sport were selected because they represent the main commercial portals through which European consumers access sports nutrition products, our sample cannot be considered fully representative of the entire European NBS market. Products distributed exclusively through specialized retailers, gym chains, pharmacy networks or direct-to-consumer brand websites may not have been captured. The findings should therefore be interpreted as describing the segment of the market with the greatest exposure to the average European online consumer, rather than the totality of available products. Search reproducibility is further constrained by the proprietary nature of the ranking algorithms of these platforms, which may be affected by sponsored placements and regional or temporal factors.

The sample size (*n* = 18 products) reflects the application of strict inclusion criteria—supplements with nitrate or beetroot juice as the sole active ingredient and with complete information available on official websites. While this approach maximizes internal validity and comparability across products, it positions the study as exploratory and descriptive in nature, and its findings should be regarded as hypothesis-generating, providing a basis for larger and more diverse follow-up research. It should also be noted that, although nitrate is an ergogenic aid with solid scientific support, relatively few brands market it as a single-ingredient supplement. Many companies incorporate nitrate or beetroot juice extract as one component among others in multi-ingredient formulations, which complicates isolated analysis of nitrate-related claims. Because our inclusion criteria were restricted to single-ingredient products, this partly explains the limited number of products identified (*n* = 18) and represents both a strength—in terms of comparability—and a constraint on the size and representativeness of the sample.

Finally, as noted in the Methods, the instrument used in this study was developed *ad hoc* and applied for the first time. Although its content validity was strengthened through expert consensus and pilot testing following Krippendorff's recommendations (27), and all coding disagreements were resolved by consensus until full agreement, no formal inter-coder reliability coefficients (Cohen's kappa, Krippendorff's alpha) were computed. Future studies applying the checklist to larger samples should adopt a parallel-coding design and report quantitative inter-rater reliability statistics. The descriptive nature of the analysis and the sample size also preclude formal inferential testing of associations between marketing characteristics and claim alignment, which represents a priority for larger follow-up studies.

## 5 Conclusions

In conclusion, nitrate- and beetroot-based dietary supplements marketed in Europe frequently present claims and usage protocols that are not fully aligned with current scientific evidence. While most products reported nitrate dosages within recommended ranges, substantial gaps were identified regarding the communication of protocols, scientific support, target sports, and contraindications.

More than half of the analyzed products relied on influencer-based marketing strategies, frequently involving athletes or public figures without demonstrable expertise in nutrition or health sciences, while none provided external scientific or institutional endorsement. This imbalance between persuasive digital communication and evidence-based information may foster misleading interpretations, particularly among populations more dependent on social media for nutrition guidance, thereby contributing to existing health inequities in the digital food environment.

These findings highlight the need to strengthen regulatory frameworks not only for supplement labeling but also for digital marketing practices and influencer-based promotion in sports nutrition, in order to better align commercial communication with current scientific consensus and to protect consumers from potentially misleading information.

## Data Availability

The datasets presented in this study can be found in the Zenodo online repository: https://zenodo.org/records/17536648.
