# Supplementary material for: From labels to influencers: evaluating the alignment of nitrate and beetroot juice claims on sports supplement marketing with scientific consensus
Source: Front Nutr. 2026 Jul 15;13:1886906. doi: 10.3389/fnut.2026.1886906 (PMC13417781; doi:10.3389/fnut.2026.1886906)
Supplement: Supplementary file 1 [file Table_1.docx]

| Table S1. Information about supplementation company and claims on their products. | | |
| --- | --- | --- |
| **Health Claims** | **Company** | **Dose** |
| Improves athletic performance | 226ERS | 400mg |
|  | Santa Madre | >500mg |
|  | AMIX | 400mg |
|  | Soccer Supplement | 400 mg |
| Improves exercise tolerance and efficiency | Soccer Supplement | 400 mg |
| Contributes to increased blood flow, boosting the supply of oxygen and nutrients to cells. | Scientific Nutrition | 495mg |
|  | Scientific Nutrition | <250mg |
|  | Scientific Nutrition | 500mg |
| Promotes muscle contraction and speeds up recovery | Scientific Nutrition | 495mg |
|  | Scientific Nutrition | <250mg |
|  | Scientific Nutrition | 500mg |
| Improves blood flow | Scientific Nutrition | 495mg |
|  | Scientific Nutrition | <250mg |
|  | Scientific Nutrition | 500mg |
|  | Beet It Sport | 428mg |
|  | Beet It Sport | 400mg |
|  | AMIX | 400mg |
|  | 6D | 500mg |
|  | Soccer Supplement | 400mg |
| Helps improve aerobic performance | Scientific Nutrition | 495mg |
|  | Scientific Nutrition | 500mg |
| Helps improve oxygen consumption efficiency and endurance | Scientific Nutrition | 495mg |
|  | Scientific Nutrition | 500mg |
|  | Beet It Sport | 428mg |
|  | Beet It Sport | 400mg |
| They will contribute to a greater supply of oxygen and nutrients to the cells. | Scientific Nutrition | 495mg |
|  | Scientific Nutrition | <250mg |
|  | Scientific Nutrition | 500mg |
| Contributes to increased athletic performance | Scientific Nutrition | 495mg |
|  | Scientific Nutrition | 500mg |
| Contributes to improving performance more efficiently | Scientific Nutrition | 495mg |
| Promotes muscle recovery | Scientific Nutrition | <250mg |
| Optimise athletic performance | Scientific Nutrition | <250mg |
| Regulates the immune system, brain, arteries, liver, pancreas, uterus, and lungs. | 226ERS | 400mg |
|  | 226ERS | 400mg |
| Increase exercise endurance | Santa Madre | >500mg |
| Improves cardiovascular health | Santa Madre | >500mg |
| Improves cognitive function in young adults | Santa Madre | >500mg |
| Improves endurance performance | BioTona | - |
|  | 6D | 500mg |
| Increases oxygen efficiency | Beet It Sport | 428mg |
|  | Beet It Sport | 400mg |
| Reduces muscle fatigue | Beet It Sport | 428mg |
|  | Beet It Sport | 400mg |
| Optimal performance for longer | Beet It Sport | 428mg |
|  | Beet It Sport | 400mg |
| Improve sprint performance | 6D | 500mg |
|  | Soccer Supplement | 400mg |
| Improve your VO2 max | UMARA | 500mg |
| Improves muscle efficiency | UMARA | 500mg |
| Improves the ability to perform repeated sprints | Soccer Supplement | 400mg |

Table S1. Health Claims and dose according with data present in the label, technical sheet and/or official website. Mg:miligrams.

**Supplementary Table S2. Operational checklist applied to each product.**

| **#** | **Variable** | **Coding options** | **Response** |
| --- | --- | --- | --- |
| **1** | Name of the supplement | Free text |  |
| **2** | Corporate brand | Free text |  |
| **3** | Authorised health claim (EFSA) | Present / Absent |  |
| **4** | Ergogenic or physiological effect in line with consensus (27) | Yes / Partially / No |  |
| **5** | Effect or claim as described by the brand (not fitting items 3–4) | Free text |  |
| **5b** | Number of declarations | Numeric |  |
| **6a** | Dose indicated by EFSA | Present / Absent |  |
| **6b** | Dose indicated by reference institutions or systematic reviews | Present / Absent |  |
| **6c** | Recommended dose stated by the company | Present / Absent |  |
| **6d** | Dose category | <300 mg / 300–500 mg / >500 mg / Not specified |  |
| **7a** | Protocol indicated by EFSA | Present / Absent |  |
| **7b** | Protocol indicated by reference institutions or reviews | Present / Absent |  |
| **7c** | Protocol stated on labelling | Present / Absent |  |
| **7d** | Protocol compliant with consensus (timing and dose) | Yes / No |  |
| **8** | Format of the product | Capsule / Pill / Powder / Liquid / Shot / Gel |  |
| **9** | Sports involved (3) | Present / Absent (specify which) |  |
| **10a** | Bibliography present | Present / Absent |  |
| **10b** | Type of article cited | Consensus / Systematic review / Meta-analysis / Other |  |
| **10c** | Year of publication | Numeric |  |
| **10d** | Reference or link accessible | Yes / No |  |
| **11** | Adverse effects described | Present / Absent (specify which) |  |
| **12** | Food safety or population restrictions | Present / Absent (specify which) |  |
| **13a** | Allergens declared | Present / Absent |  |
| **13b** | List of allergens | Free text |  |
| **14a** | Celebrity or influencer on the website or product labelling | Present / Absent |  |
| **14b** | Does the influencer have training or experience with the supplement’s content? | Yes / No / Not applicable |  |
| **15** | External endorsement (scientific institution or association) | Present / Absent (specify which) |  |
| **16** | Anti-doping seal | Present / Absent (specify which) |  |
| **17** | Patented ingredients | Present / Absent (specify which) |  |
| **18** | Scientific advice disclosed (staff or collaborators) | Present / Absent (specify who) |  |

***Notes:*** *Operational version of the ad hoc checklist applied independently to each product by two coders. Most variables were coded dichotomously (presence/absence) to minimise subjective interpretation; the classification variables (items 4, 6d and 7d) followed the operational criteria described in the footnotes of Tables 2a and 2b. Numbers in parentheses refer to the supporting references used to define each variable, consistent with Table 1. Items marked as “specify which/who” allowed the coders to record the corresponding free-text detail when the variable was present.*
